# The concept of broad exposure facilitates uniportal video-assisted thoracoscopic mediastinal lymph nodes dissection

**DOI:** 10.1186/s13019-021-01519-6

**Published:** 2021-05-21

**Authors:** Wei Wang, Sunyin Rao, Mingsheng Ma, Yunchao Huang, Guangqiang Zhao, Xin Cui, Qinling Sun, Lianhua Ye

**Affiliations:** 1 Department of Thoracic Surgery, The Third Affiliated Hospital of Kunming Medical University, No. 519 Kunzhou Road, Xishan District, Kunming City, Yunnan Province China; 2 Department of Thoracic Surgery, Taihe Hospital (Hubei University of Medicine), Shiyan, China; 3 Department of Thoracic Surgery, The Sixth Affiliated Hospital of Kunming Medical University, Yuxi, China

**Keywords:** Broad exposure, Mediastinal lymph nodes dissection, Uniportal, Video-assisted thoracoscopic surgery, Non-small cell lung cancer

## Abstract

**Background:**

Systematic lymph node dissection is an important part of radical resection for lung cancer. Insufficient incision of the mediastinal pleura results in a tapered or tunnel-like operation surface, which increases the difficulty of uniportal video-assisted thoracoscopic mediastinal lymph node dissection. The objective of this study was to report our concept of broad exposure and investigate the efficacy and safety of this concept in uniportal video-assisted thoracoscopic mediastinal lymph nodes dissection.

**Methods:**

We retrospectively analyzed the clinical data of the 204 non-small cell lung cancer patients who underwent uniportal video-assisted thoracoscopic surgery for anatomical lobectomy and systematic lymph node dissection following the concept of broad exposure. SPSS 23.0 software was used for statistical analysis.

**Results:**

All operations were completed under uniportal video-assisted thoracoscopic surgery following the concept of broad exposure. The median surgery time was 102 (range, 76–285) minutes and the median blood loss was 50 (range, 20–900) milliliters. The median chest tube duration time was 2 (range, 1–6) days, the median postoperative hospital duration time was 5 (range, 4–10) days. The median number of dissected lymph node stations and dissected lymph nodes were 8 (range,6–9) and 15(range,12–19), respectively. The median number of dissected mediastinal lymph nodes stations and dissected mediastinal lymph nodes were 5(range,3–6) and 11(range,10–15), respectively. The up-staging rate of N staging was 6.86%. The postoperative complication rate was 10.29% and there was no perioperative death.

**Conclusions:**

According to our results, it’s effective and safe to perform uniportal video-assisted thoracoscopic mediastinal lymph nodes dissection following the concept of broad exposure. This new concept not only emphasizes sufficient exposure, but also focuses on protection of important tissues.

## Introduction

At present, lung cancer is the malignant tumor with the highest morbidity and mortality [1]. Surgery is the best treatment for early stage lung cancer. Video-assisted thoracoscopic surgery (VATS) for radical resection of lung cancer has been becoming the mainstream method of lung cancer surgery in recent years. The development of uniportal VATS technology brings a more minimally invasive treatment method for lung cancer patients. Systematic lymph node dissection, an important part of radical resection of lung cancer is one of the main factors affecting the long-term prognosis [2]. Some studies believe that uniportal VATS for radical resection of lung cancer can achieve similar surgical results as traditional triportal VATS [3, 4].

However, the fluency of the uniportal VATS is seriously affected by the mutual interference of instruments used by the operator and the assistant. The limited exposure of the surgical field seriously affects the safety of operation and the integrity of mediastinal lymph nodes dissection (MLND). MLND is challenging for uniportal VATS radical resection of lung cancer, the key lies in how to effectively expose the mediastinal lymph nodes (MLNs). Insufficient incision of the mediastinal pleura results in a tapered or tunnel-like operation surface, which increases the difficulty of uniportal video-assisted thoracoscopic mediastinal lymph node dissection. We summarized the experience of uniportal video-assisted thoracoscopic MLND in recent years and proposed the concept of broad exposure. The objective of this study was to report the concept of broad exposure and investigate the efficacy and safety of this concept in 204 patients who underwent uniportal video-assisted thoracoscopic MLND.

## Materials and methods

### Patients

A total of 204 patients with non-small cell lung cancer (NSCLC) underwent uniportal VATS for anatomical lobectomy and systematic lymph node dissection following the concept of broad exposure in the department of thoracic surgery, the Third Affiliated Hospital of Kunming Medical University between August 2017 and February 2020. We retrospectively analyzed the clinical data of the patients. The inclusion criteria for the patients were: (1) anatomical lobectomy and systematic lymph node dissection were completed with uniportal VATS following the concept of broad exposure; (2) the dissected stations of MLNs > 3, which must include the subcarinal station, and total number of dissected MLNs > 10; (3) postoperative pathology was NSCLC. The exclusion criteria were as follows: (1) intraoperative conversion to thoracotomy; (2) intraoperative investigation revealed MLNs calcification or hilar freezing; (3) the patient has severe coronary artery stenosis, severe arrhythmia and cardiopulmonary insufficiency before surgery. The characteristics of the patients were shown in Table 1. This study was approved by the institutional review committee and ethics committee of the Third Affiliated Hospital of Kunming Medical University (NO.KY2020201) and individual consent for this retrospective analysis was waived.

**Table 1:** Patients’ characteristics

Characteristic	Total *N* = 204
Age (y)	
Range	34–75
Median	54
Male/female	101/103
Smoking / non-smoking	69/135
Comorbidity	
Diabetes	5
Hypertension	8
Coronary heart disease	6
Chronic obstructive pulmonary diseases	4
Others	10
History of malignancy	
Thyroid cancer	3
Breast cancer	2
Cervical cancer	1
Rectal cancer	1
Clinical TNM staging	
IA	36(17.6%)
IB	54(26.4%)
IIA	62(30.4%)
IIB	33(16.2%)
IIIA	15(7.4%)
IIIB	4(2.0%)
Clinical N staging	
N0	172(84.3%)
N1	19(9.3%)
N2a	13(6.4%)

### Surgical techniques

All patients received double lumens endotracheal intubation, combined intravenous and inhalation anesthesia. The unilateral lung of the disease-free side was ventilated during operation. All patients were placed in the lateral decubitus position, both the surgeon and the endoscopic assistant stood on the ventral side of the patient, or endoscopic assistant stood on the dorsal side of the patient. A 3-cm incision was made in the 4th or 5th intercostal space of the mid-axillary line, and the wound protector was inserted. Thoracoscopy and operating instruments were all through the wound protector, and an appropriate surgical resection method was designed based on the exploration of the lesion and the preoperative imaging characteristics.

The concept of broad exposure was as opposed to an inadequate incision of the mediastinal pleura. According to the concept of broad exposure, the inferior pulmonary ligament was loosened first, then the anterior and posterior mediastinal pleura was fully cut open to allow the MLNs to be visually exposed to the operative surface rather than through a narrow space. The range of mediastinal pleural incision was as follows: anterior to the phrenic nerve, posterior to the vagus nerve, up to the level of the azygos arch (right side) or the aortic arch (left side), and down to the level of the inferior pulmonary veins. The assistant pushed the lung towards the opposite direction away from the operating area with a sponge forceps to avoid interference between the instruments in the narrow operating space. The surgical table was adjusted at the same time, and the deeper MLNs became superficial with the help of the gravity of the tissue itself, so as to achieve the best exposure effect. The concept of broad exposure improved the effect of exposure for stations 2R, 4R, 4 L, 5, 6, and 7 lymph nodes during uniportal VATS.

### Techniques specific to right-sided stations 2R and 4R MLND

**Fig. 1:**
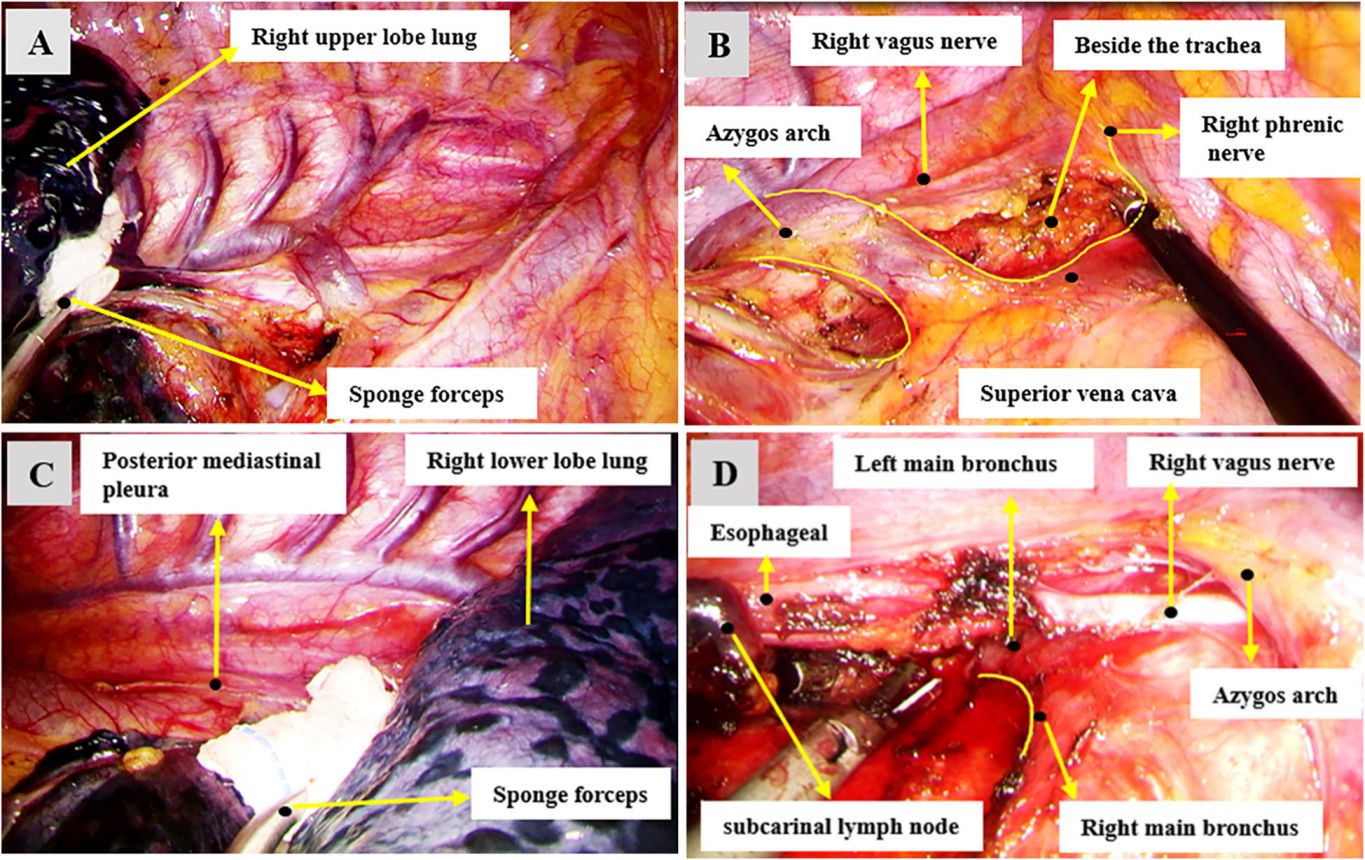
Examples of exposure effect of right-sided mediastinal lymph node dissection. **a** The right upper lobe lung was pushed to the foot and dorsal side of the patient by the assistant with a sponge forceps for better exposure of the stations 2R and 4R lymph nodes. **b** The stations 2R and 4R lymph nodes and adipose tissue in the upper paratracheal area were removed after the azygos arch was completely naked. **c** In order to get a better exposure of the right-sided subcarinal lymph nodes, the right lower lobe lung was pushed towards the sternum by the assistant with a sponge forceps. **d** An example of the effect of the right-sided subcarinal lymph nodes exposure and the adjacent anatomical structure. The posterior mediastinal pleura was completely cut open, the lymph nodes were separated from the esophagus, right main bronchus, pericardium and left main bronchus

When removing the stations 2R and 4R lymph nodes, the surgical table was adjusted to head high and foot low, the assistant pushed the right upper lobe lung to the foot and dorsal side (Fig. 1a). The lymph nodes and adipose tissue around the lower paratracheal were completely removed through the azygos arch. Pulled the fascia near the right vagus nerve backward, continued to cut open the mediastinal pleura along the superior border of the azygos arch and the right posterior border of the superior vena cava. The lymph nodes and adipose tissue in the upper paratracheal area were removed after the azygos arch was completely naked (Fig. 1b).

### Techniques specific to right-sided subcarinal (station 7) MLND

The surgical table was tilted 15 ° - 30 ° to the ventral side, and the assistant pushed the right lower lobe lung towards the sternum (Fig. 1c). The posterior mediastinal pleura had been completely cut open, the lymph nodes were separated from the esophagus, the right main bronchus, the pericardium and the left main bronchus, the lymph nodes and surrounding adipose tissue were completely removed (Fig. 1d).

### Techniques specific to left-sided stations 5 and 6 MLND

**Fig. 2:**
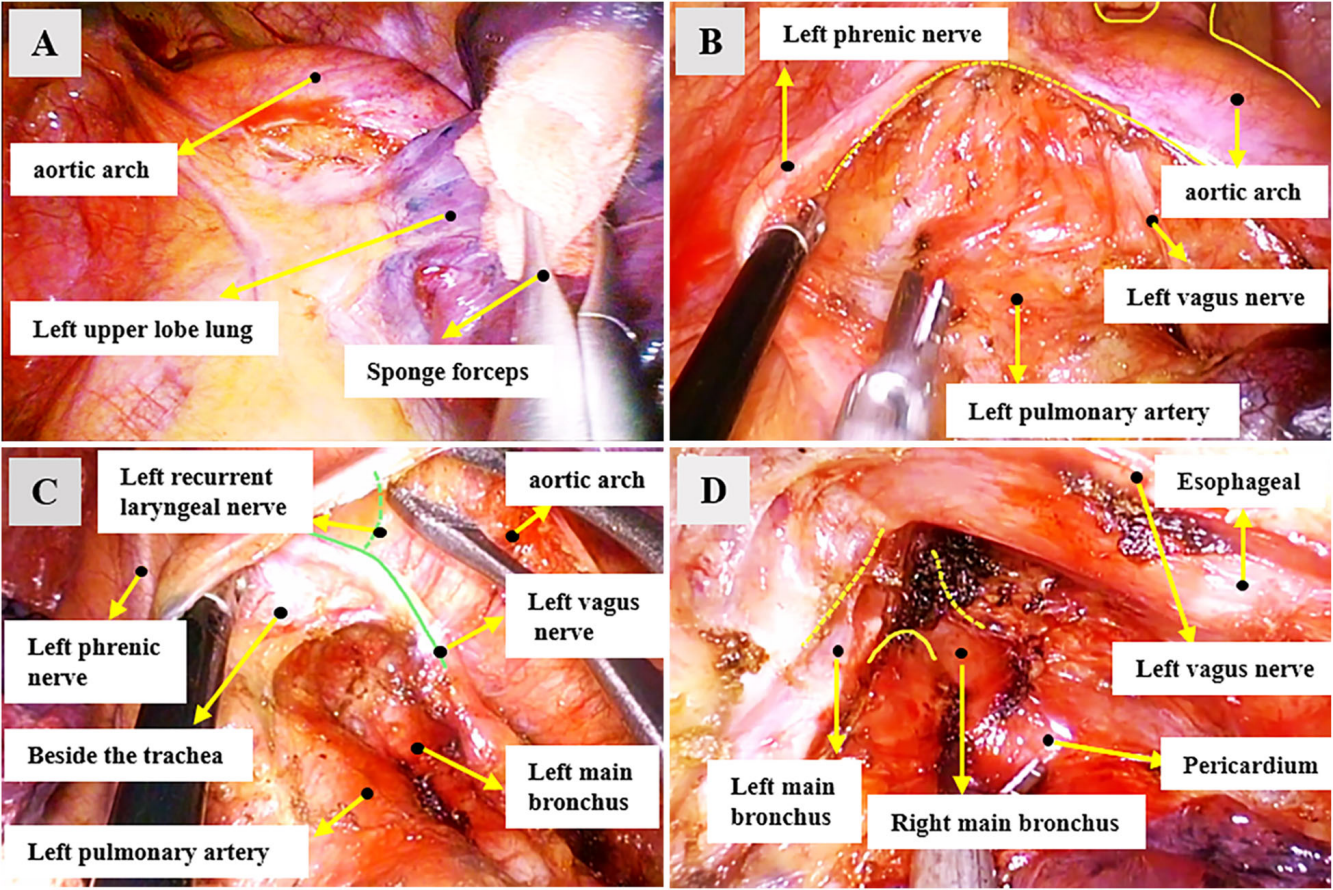
Examples of exposure effect of left-sided mediastinal lymph node dissection. **a** The left upper lobe lung was pushed towards the diaphragm and spine by the assistant with a sponge forceps when removing the stations 5 and 6 lymph nodes. **b** An example of resection of stations 5 and 6 lymph nodes, they were picked up with a suction and pushed to the dorsal side, then were separated from the aortic arch, arterial ligament, and pulmonary artery trunk. **c** Protection of left vagus nerve and left recurrent laryngeal nerve during the resection of station 4 L lymph node. **d** An example of the effect of the left-sided subcarinal lymph nodes exposure and the adjacent anatomical structure

When removing the stations 5 and 6 lymph nodes, the assistant pushed the lung towards the diaphragm and spine (Fig. 2a). We generally cut the mediastinal pleura along the triangular area formed by the back of the phrenic nerve, the front of the vagus nerve and the left pulmonary artery to expose the station 5 lymph nodes. The lymph nodes were picked up with a suction device, and the surrounding fatty tissues were removed together. Then cut the mediastinal pleura along the front of the phrenic nerve, and pulled the phrenic nerve and the surrounding fascia back to expose the station 6 lymph nodes. Alternatively, at the same time that station 5 lymph nodes were removed, the station 6 lymph nodes can be exposed from below the phrenic nerve and remove them together. (Fig. 2b).

### Techniques specific to left-sided stations 4 L MLND

When the stations 4 L lymph nodes were removed, the surgical table was adjusted to head high and foot low. The assistant pushed the left upper lobe lung to the foot and ventral side, then pulled back the fascia near the left vagus nerve and the left recurrent laryngeal nerve. The lymph nodes and adipose tissue around the lower para-trachea were fully dissociated under the aortic arch (Fig. 2c).

### Techniques specific to left-sided subcarinal (station 7) mediastinal lymph nodes dissection (MLND)

The surgical table was tilted 15 °-30 ° to the ventral side, and the assistant pushed the left lower lobe lung towards the sternum. The posterior mediastinal pleura was completely cut open, then the descending aorta and esophagus were pushed with curved suction. The lymph nodes were separated from the esophagus, left main bronchus, pericardium and right main bronchus, finally were completely resected with surrounding adipose tissue (Fig. 2d).

### Statistical analysis

SPSS 23.0 software was used for statistical analysis. Numerical variables that conformed to normal distribution were presented as mean ± standard deviation, numerical variables that were not normally distributed were presented as median (minimum-maximum). The number and proportion were counted for classification variables.

## Results

All operations were successfully completed under uniportal VATS following the concept of broad exposure, including 193 cases of lobectomy, 8 cases of bilobectomy, 3 cases of pneumonectomy. The perioperative results were shown in Table 2. The median surgery time was 102 min (range, 76–285 min), and the median blood loss was 50 ml (range, 20–900 ml). The median chest tube duration time was 2 days (range, 1–6 days), and the median postoperative hospital duration time was 5 days (range, 4–10 days). The final pathological examination revealed that all patients had radical resection with negative margins, 121 cases of adenocarcinoma, 64 cases of squamous cell carcinoma, and 19 cases of other types of non-small cell lung cancer. The median number of dissected lymph node stations was 8(6–9), the median number of dissected lymph nodes was 15(12–19), the median number of dissected MLNs stations was 5(3–6), the median number of dissected MLNs was 11(10–15). The up-staging rate of N staging was 6.86%(3 patients upgraded from cN0 to pN1, 7 patients upgraded from cN1 to pN2a, 4 patients upgraded from cN2a to pN2b). The postoperative complication rate was 10.29% (21/204, including 1 patient with chylothorax, 2 patients with arrhythmia, 1 patient with hoarse voice, 6 patients with atelectasis, 9 patients with lung infection and 2 patients with others). All patients had no obvious symptoms when they left hospital, and there was no perioperative death. No recurrence or death was observed within 13 months of the median follow-up (range, 1–30 months), and the last follow-up was until March 2020.

**Table 2:** Perioperative clinical results of patients

Variables	Total *N* = 204
Surgical resection of lung cancer	
Lobectomy	193
Bilobectomy	8
Pneumonectomy	3
Operative time (min)	102(76–285)
Blood loss (mL)	50(20–900)
Number of dissected lymph node stations	8(6–9)
Number of dissected lymph nodes	15(12–19)
Number of dissected MLNs stations	5(3–6)
Number of dissected MLNs	11(10–15)
Postoperative drainage duration (d)	2(1–6)
Postoperative hospital duration(d)	5(4–10)
Postoperative complications	
Lung infection	9(4.4%)
Atelectasis	6(2.9%)
Chylothorax	1(0.5%)
Arrhythmia	2(1%)
Voice hoarse	1(0.5%)
Others	2(1%)
Pathologic types	
Squamous cell carcinomas	64(31.4%)
Adenocarcinoma	121(59.3%)
Others	19(9.3%)
Pathological N staging of lung cancer	
N0	173(84.8%)
N1	12(5.9%)
N2a	15(7.3%)
N2b	4(2.0%)
Up-staging of N staging	
cN0 to pN1	3(1.5%)
cN1 to pN2a	7(3.4%)
cN2a to pN2b	4(2.0%)

## Discussion

Since 2004, uniportal VATS was first reported for biopsy of pulmonary nodules, its application scope has been gradually expanded for lobectomy, segmentectomy, vascular reconstruction and bronchoplasty with the improvement of endoscopic instruments and surgical techniques [5–7]. Some studies believed that uniportal VATS for lung cancer radical surgery had advantages in controlling postoperative pain, reducing postoperative chest tube duration, shortening postoperative hospital duration, and had the same effect of lymph node dissection compared with triportal VATS [8]. Because MLND is an important part of radical resection for lung cancer, the integrity of MLND under uniportal VATS is a key factor in determining whether radical resection of tumor can be achieved. Some limitations of uniportal VATS, such as mutual interference between instruments, insufficient exposure, and high coordination requirements between surgeon and assistant [9], these all increase the difficulty and surgical risk of uniportal video-assisted thoracoscopic MLND.

In our study, uniportal video-assisted thoracoscopic MLND was performed following the concept of broad exposure. A wide range of incision of the mediastinal pleura relieves the mediastinum pleura from covering the MLNs, and increases the mobility of the lung and hilar tissue. The assistant pushes the lung lobe in a certain direction away from the MLNs, so that the deeper MLNs become superficial. The sufficient isolation for the target area of the operation with suction device and energy equipment, and the appropriate skeletalization of the surrounding structure makes it easier to achieve complete dissection of the MLNs. Our results showed that the total number of dissected lymph nodes and dissected lymph nodes stations were 15(12–19) and 8(6–9), respectively; the number of dissected MLNs and dissected MLNs stations were 11(10–15) and 5(3–6), respectively. We strictly followed the principle of complete lymph node resection,lymph node fragmentation was inevitable, the reported number of lymph nodes was based on post-operative pathology reports. The uniportal video-assisted thoracoscopic MLND following the concept of broad exposure meets the requirements of radical resection of lung cancer [10], at least three stations of MLNs be dissected, subcarinal lymph nodes must be included, and the number of dissected MLNs must not be less than 10.

The uniportal video-assisted thoracoscopic MLND following the concept of broad exposure reduces the instruments in the surgical operation area, avoids the mutual interference between instruments to the greatest extent, reduces the surgeon’s dependence on assistants, and increases the cooperation between the surgeon and the assistant. According to our results, there were no perioperative deaths, the median operation time (102 min)and the median intraoperative blood loss (50 ml) were comparable to the data reported in another study [11]. The safety of video-assisted thoracoscopic MLND following the concept of broad exposure was confirmed by our results. When exposure is insufficient, it is easy to cause damage to the lymphatic vessels and vagus nerve branches. The risk of conventional tunneling lymph node dissection was reduced by exposure to large planes.

It’s difficult to expose the operating area during the process of uniportal video-assisted thoracoscopic MLND, especially the subcarinal lymph node located in the posterior mediastinum. There are many important organs around the carina and the operation space is narrow. At present, most surgeons use the method of pulling the lung forward to expose the subcarinal area [12], which may cause lung damage. The concept of broad exposure emphasizes the protection of important organizational structures while exposure to large planes. Use sponge forceps to push lung tissue instead of directly clamping and pulling to reduce lung tissue damage. The device should be used to block the nerve to the side or lift the fascia near it, rather than directly pull the nerve to move it away from the operation area. Our results showed that the incidence of postoperative complications, median postoperative drainage duration and median postoperative hospital duration were 10.29%, 2 days and 5 days, respectively. There was no increase in tissue damage due to broad exposure compared with the data reported by Cai et al. [13]. So broad exposure requires not only adequate exposure, but also the protection of important organs.

This study has some limitations. First, this is a single-center retrospective study that may have a selection bias; second, there is a lack of long-term follow-up of the included patients. However, broad exposure is a novel concept proposed by our center for uniportal video-assisted thoracoscopic MLND, it may provide new surgical ideas for thoracic surgeons. We plan to conduct a prospective comparative study within multiple centers in the future, and observe the long-term effects of the patients, to further investigate the advantages of uniportal video-assisted thoracoscopic MLND following the concept of broad exposure.

## Conclusions

According to our results, it’s effective and safe to perform uniportal video-assisted thoracoscopic MLND following the concept of broad exposure. This new concept emphasizes sufficient exposure, which makes it easier to achieve complete dissection of MLNs, while focuses on protection of important tissues. Its long-term efficacy needs to be confirmed by further follow-up.

## Data Availability

The datasets generated and analyzed during the current study are available from the corresponding author on reasonable request.

## Abbreviations

VATS: Video-assisted thoracoscopic surgery
MLND: Mediastinal lymph nodes dissection
MLNs: Mediastinal lymph nodes
NSCLC: Non-small cell lung cancer
